# Xylanase impact beyond performance: A microbiome approach in laying hens

**DOI:** 10.1371/journal.pone.0257681

**Published:** 2021-09-20

**Authors:** Veerle Van Hoeck, Ingrid Somers, Anas Abdelqader, Alexandra L. Wealleans, Sandy Van de Craen, Dany Morisset

**Affiliations:** 1 Animal Nutrition and Health, Kemin Europa N.V., Herentals, Antwerp, Belgium; 2 Department of Animal Production, The University of Jordan, Amman, Jordan; Tokat Gaziosmanpasa Universitesi, TURKEY

## Abstract

Anti-nutritional compounds such as non-starch polysaccharides (NSP) are present in viscous cereals used in feed for poultry. Therefore, exogenous carbohydrases are commonly added to monogastric feed to degrade these NSP. Our hypothesis is that xylanase not only improves laying hen performance and digestibility, but also induces a significant shift in microbial composition within the intestinal tract and thereby might exert a prebiotic effect. In this context, a better understanding on whether and how the chicken gut microbial population can be modulated by xylanase is required. To do so, the effects of dietary supplementation of xylanase on performance, apparent total tract digestibility (ATTD) and cecal microbiome in laying hens were evaluated in the present study. A total of 96 HiSex laying hens were used in this experiment (3 diets and 16 replicates of 2 hens). Xylanase was added to the diets at concentrations of 0, 45,000 (15 g/t Xygest^TM^ HT) and 90,000 U/kg (30 g/t Xygest HT). The diets were based on wheat (~55%), soybean and sunflower meal. The lowest dosage, 45,000 U/kg, significantly increased average egg weight and improved feed efficiency compared to the control treatment (P<0.05). Egg quality parameters were significantly improved in the experiment in response to the xylanase addition. For example, during the last 28 days of the trial, birds receiving the 45,000 U/kg and the 90,000 U/kg treatments exhibited an increase in Haugh units and albumin heights (P<0.05). Compared with the control, the ATTD of organic matter and crude protein were drastically improved in the 45,000 U/kg treatment group (P<0.05). Furthermore, gross energy and the ATTD of crude fat were improved significantly for birds fed 90,000 U/kg group compared to the control. Importantly, 16S rRNA gene analysis revealed that xylanase at 45,000 U/kg dosage can exert a change in the cecal microbiome. A significant increase in beneficial bacteria (*Bacilli* class; *Enterococcaceae* and *Lactobacillales* orders; *Merdibacter*, *Enterococcus* and *Nocardiopsis* genera; *Enterococcus casseliflavus* species) was documented when adding 45,000 U/kg xylanase to the diet of laying hens. In conclusion, dietary supplementation of xylanase 45,000 U/kg significantly improved laying hen performance and digestibility. Furthermore, microbiome data suggest that xylanase modulates the laying hen bacterial population beneficially, thus potentially exerting a prebiotic effect.

## Introduction

Reducing the costs of feed production is a challenging issue for the poultry industry. Due to recent increases in grain prices, and general long-term price volatility, the animal feed industry has been working towards lowering nutritional costs [1]. Wheat has become an important source of energy in poultry diets, even in markets that do not traditionally rely on wheat, due to shortages in corn supply and the increase in corn price. Poultry do not produce endogenous carbohydrases capable of hydrolyzing the NSPs, such as arabinoxylans, present in viscous cereals [2]. NSP can decrease the bird’s ability to efficiently utilize protein and energy from these feed materials [3].

For this reason, the use of exogenous enzymes to improve poultry performance is widespread and still increasing [4], and enzyme supplementation of wheat diets is a common practice in commercial poultry nutrition [5]. Dietary supplementation of exogenous enzymes is proven to be an effective cost-saving mechanism [1]. Exogenous enzymes can hydrolyze NSP, which facilitates the digestibility and utilization of nutrients. NSP-hydrolysing enzymes contribute to more than 50% of the global feed enzyme market, with more xylanases sold than β-glucanases. Xylanase is supplemented to commercial wheat-based compound feeds for poultry in order to degrade the polysaccharide cage structures around nutrients. Xylanases are the major enzymes involved in arabinoxylan degradation, hydrolyzing the 1,4-β-D-xylosidic linkage between xylose residues in the backbone in a random manner [6].

Supplementation of xylanase facilitates animal performance, improves absorption of feed components, and increases the efficiency of meat and egg production [7, 8]. The addition of xylanases in wheat-based diets can lower intestinal viscosity via the partial hydrolysis of wheat NSPs [9]. As stated by Zhang et al. [9], the latter is reflected in improvements to nutrient digestibility [10, 11]. Xylanase supplementation can also improve immunity [11] and can protect the chickens against *Clostridium perfringens* [12] and *Salmonella typhimurium* infections [13].

Securing a balance between beneficial and pathogenic bacterial populations in the intestinal tract is critical for the health of the animal [14]. Kogut et al. [15] documented that the chicken gastrointestinal tract is home to a complex microbial population that underlines the links between diet and health. This microbiome flexibility provides an attractive tool for future potential approaches to improve intestinal health in chickens. However, a better understanding of how any intervention, such as xylanase addition, can modulate intestinal populations is needed as poultry producers are encouraged to reduce use of antibiotics, both prophylactic and therapeutic. As such, cost-effective alternatives to antibiotics to tackle these microbial pathogens in chickens would be of great value to the food industry and to the consumer [16].

In the present study, the role of xylanase as modulators of the microbiome has been characterized. The study hypothesis was that xylanase addition to the laying hen diet would both increase performance and digestibility. Furthermore, it was proposed that the xylanase impacts on the gut microbial population and thereby serves as a prebiotic. Cecal samples were collected from laying hens supplemented with xylanase and were compared to cecal samples obtained from control animals for microbiome analysis using sequence variation in the 16S rRNA gene, which is widely used to characterize diverse microbial communities (for review [17]).

## Materials and methods

### Animal experiments

The procedures related to animal care used in this experiment were approved by the Animal Care and Use Committee of the University of Amman (Jordan). Animals were reared and treated in compliance with the EU Directive 2010/63/EU covering the protection of the animals used for experimental or other scientific purposes.

#### Xylanase product

Xygest^TM^ HT is an intrinsically thermostable monocomponent xylanase produced by *Thermopolyspora flexuosa* expressed in *Pichia pastoris* and is a beta 1–4, endo-xylanase enzyme belonging to the GH11 family, designed to improve the degradation of dietary fiber to maximize energy utilization of the diet. Xygest HT has a minimal xylanase activity of 3,000,000 U/g on a corn starch-based carrier.

### Experimental design

A laying hen trial was carried out to investigate the effects of the dietary addition of xylanase on the performance (egg production, egg weight, egg mass and feed conversion efficiency), digestibility and cecal microbiome in HiSex laying hens from 22 weeks of age under research farm conditions. The trial lasted for 84 days. A total of 96 laying hens were used in this experiment (3 diets, 16 replicates, 2 birds per replicate). Xylanase was added to the diets at concentrations of 0, 45,000, and 90,000 U/kg. The diets were based on wheat (~55%), soybean and sunflower meals. A full overview on diet composition is presented in Tables 1 and 2.

**Table 1:** Composition of the basal diet.

Wheat soft	55.03%
Soybean meal 47% CP	15.40%
Sunflower meal 34% CP	10.00%
Limestone	9.08%
Corn yellow	5.00%
Soybean oil	3.73%
Mono calcium phosphate	0.55%
Premix-min-vit layer	0.40%
Sodium chloride	0.29%
D.L. methionine	0.16%
L-Lysine HCL	0.15%
Phytase*	0.06%
Sodium bicarbonate	0.05%
Pigment red	0.04%
L-threonine	0.04%
L-valine	0.02%
**Total**	**100.00%**

*Quantum Blue 5G, AB Vista, Marlborough, UK, providing 500 FTU/kg.

CP: Crude protein.

**Table 2:** Calculated analyses of the basal diet.

Dry Matter	89.00%
Crude Protein	17.50%
Crude Fat	4.99%
Crude Fiber	3.99%
NSP	14.88%
Starch	35.83%
Calcium	3.90%
Phosphorus	0.53%
Dig. Phosphorus	0.40%
Sodium (Na)	0.17%
Chloride	0.25%
Potassium (K)	0.73%
Lysine	0.76%
Methionine	0.41%
Cysteine	0.26%
Methionine and Cysteine	0.67%
Tryptophan	0.18%
Threonine	0.53%
Isoleucine	0.61%
Arginine	1.00%
Valine	0.70%
AME	2,700.00 kcal/kg

AME: Apparent metabolizable energy.

The different treatments are depicted in Table 3. The treatments were presented as mash feed, and feed and water were available *ad libitum* throughout the experiment. Bird health was examined daily in the pens and any variation in appearance and/or behavior was recorded. Mortality was monitored daily, every morning. Before the start of the trial, feed samples from each treatment were provided to Kemin Europa N.V. for recovery analysis of the xylanase. For the determination of the xylanase activity in feed, a slightly modified version of a commercially available test kit (Xylazyme AX Tablets from Megazyme International Ireland) was used. The aqueous feed extracts were incubated with the Xylazyme tablets at pH 4.8 using the citrate-phosphate buffer for 15 min at 50°C. The results showed (Table 3 –measured inclusion level) that the xylanase had been added correctly to the diets according to specifications in the trial protocol.

**Table 3:** Enzyme activity for the different treatments.

Treatment	Dosage		Intended inclusion level	Measured inclusion level
T1	0 g/t Xygest HT	Control	0 U/kg	0 U/kg
T2	15 g/t Xygest HT	Minimal recommended dosage laying poultry	45,000 U/kg	42,514 U/kg
T3	30 g/t Xygest HT	Highest recommended dosage laying poultry	90,000 U/kg	111,854 U/kg

Note: Unit definition = 1 unit of activity is the amount of enzyme that liberates 1 μg of xylose equivalents per minute and per gram of enzyme product.

### Measurements and records

#### Xylanase product

*Visualization of wheat bran treated with Xygest HT*. To test the *in vitro* effectivity of the current xylanase degrading wheat bran into smaller fractions, scanning electron microscope (SEM; EM-30, Coxem, Daejeon, Korea & Sputter, ion-coater, STP-20, Coxem, Daejeon, Korea) visualization was performed on wheat bran. Wheat bran was incubated for 24 hours at 40°C and pH 5.0 without or with Xygest HT exposure and then visualized.

#### Performance

(i) Body weight: Individual body weight was measured at the start of the trial period at 22 weeks of age and at the end of the trial. (ii) Egg production: All eggs laid were collected and recorded on a daily basis (per pen replication). Rate of lay (% lay) was calculated as number of eggs collected divided per number of hens in the cage x100. (iii) Egg weight, egg mass and classification: All eggs laid were collected and weighed as an average per pen on a daily basis. Egg mass was calculated lay % production multiplied by average egg weight and divided by 100. Eggs were commercially classified based on egg weights. (iv) Feed intake and feed efficiency: Feed intake was calculated as average feed intake per hen per day (g/hen/day). Feed intake was corrected for mortality based on number of hens per cage. Feed efficiency was then calculated as g feed/g of egg mass. (v) Egg quality parameters: All eggs laid from weeks 4, 8 and 12 of the trial were used for the egg yolk color evaluation using the DSM fan method. More specifically, on the last week of each period (namely at week 4, week 8 and week 12), data were collected for the entire week (for 7 consecutive days, each 24-hour period). At the end of the week, the average was taken of the 7 days. The proportion of dirty and cracked eggs was recorded on daily basis. Haugh units for an individual egg were calculated as: Haugh units (HU) = 100 × log (h − 1.7 × w^0.37^ + 7.6), where h and w are the measured albumen height (mm) and intact egg weight (g), respectively [18]. At week 12, eggs were commercially classified based on egg weights. All eggs laid in a 24-hour period were collected, weighed individually and classified commercially (S, M, L, XL), where S < 52.5 g; 52.5 g≤ M < 62.5 g: 62.5 g ≤ L< 72.5 g; XL > 72.5 g. (vi) Digestibility: On day 23 of the experiment, a total of 36 hens (12 hens from each treatment) were selected. Selection was performed so that each selected hen was very close to the average body weight of the flock, and hens with the highest deviation from the average were excluded. Each hen was allocated to an individual cage. Titanium dioxide (TiO_2_) was included in all experimental diets as an indigestible marker to determine the apparent total tract digestibility (ATTD) of dry matter (DM), organic matter (OM), nitrogen (N), crude fat, crude fiber, and gross energy (GE). TiO_2_ was included in the same experimental diets at a concentration level of 5 g/kg. The apparent digestibility trial consisted of 5 days of pre-collection period for adaptation purposes (days 23 to 27 of the experiment) and 4 days of excreta collection period (days 28 to 31 of the experiment). Excreta samples were collected from each individual cage (replication). Excreta samples were oven-dried at 60°C for 72 h, after which diets and excreta samples were ground to pass through a 0.5 mm screen using a mill grinder (Retsch ZM 100, Retsch GmbH and Co., K.G., Haan, Germany). Diets and excreta samples were analyzed for dry matter (AOAC method 930.15), organic matter (AOAC method 942.05), crude fiber (AOAC method 962.09), and crude fat (AOAC method 920.39) according to the methods of the Association of Official Analytical Chemists [19] procedures. For crude protein digestibility, excreta were analyzed for N contents using micro-Kjeldahl method (AOAC method 988.05). Crude protein digestibility was calculated as follows: N_retention = N per g diet–N per g excreta ×(TiO_2_diet/TiO_2_excreta), where N_retention = g N retained by the hen per g of diet. N retention was corrected for the content of uric acid nitrogen in the excreta. Uric acid nitrogen was determined according to Marquardt [20] and subtracted from the excreta nitrogen. GE was determined by measuring the heat of combustion in the samples, using a bomb calorimeter (IKA®–WERKE, C5001, Germany). The indigestible marker TiO_2_ was analyzed via UV absorption spectrophotometry (UVIKON 810, Tegimenta, Rotkreuz AG, Switzerland), according to the methods described by Short *et al*. [21] and Myers *et al*. [22]. The ATTD of both methods were then calculated using the formulas presented by Smeets *et al*. [23]. The apparent metabolizable energy corrected for nitrogen (AMEn) was calculated by correction for zero nitrogen retention by assuming 8.22 kcal/g nitrogen retained in the body [24].

#### Beyond performance

*Microbiome characterization*. Cecal samples from 16 laying hens were screened for microbiome. For this, only cecal samples from the control treatment and the 45,000 U/kg treatment were collected and analyzed (8 repeats per treatment). The 16S rRNA sequence analysis was used for reconstruction of bacterial phylogenies and classifications. With the continuous development of high-throughput sequencing platforms, the Illumina sequencing platform can realize the PE250 strategy sequencing. For taxonomic classification, it is enough to sequence individual hypervariable regions instead of the entire gene [25, 26]. In short, the methods used are the following: (i) Cecal sampling: At the end of the performance trial, 8 hens from the control group and 8 hens from the 45,000 U/kg treatment group were euthanized and removal of the gastrointestinal tract was performed. Cecal content was squeezed in Eppendorf tubes and stored at -80°C until extraction. (ii) DNA extraction: QIAamp PowerFecal Pro DNA Kit (Qiagen) was used to extract DNA from the cecal samples. Briefly, 250 mg of cecal content was placed in a sterile, round-bottom 2 mL tube containing 1.4 mL lysis buffer and the remainder of the protocol was followed as described by the manufacturer. (iii) Amplicon Generation as presented in Table 4.

**Table 4:** Amplicon generation.

Region:	Bacterial 16S V3-V4
Fragment Length:	466bp
Primers:	341F: CCTAYGGGRBGCASCAG
	806R: GGACTACNNGGGTATCTAAT

All PCR reactions were carried out in 30 μL reactions with 15 μL of Phusion® High-Fidelity PCR Master Mix (New England Biolabs), 0.2 μM of forward and reverse primers, and about 10 ng template DNA. Thermal cycling is started with the initial denaturation at 98°C for 1 min, followed by 30 cycles of denaturation at 98°C for 10 s, annealing at 50°C for 30 s, and elongation at 72°C for 60 s. The final step was 72°C for 5 min. (iv) PCR Products quantification and qualification: Mixing equal volume of 1X loading buffer (contains SYBER green) with PCR products and operate electrophoresis on 2% agarose gel for detection. Samples with one bright main strip between 400–450 bp were chosen for further experiments. (v) PCR Products Mixing and Purification: PCR products were mixed in equidensity ratios. Then, the mixed PCR products were purified with GeneJET Gel Extraction Kit (Thermo Scientific). (vi) Library preparation and sequencing: Sequencing libraries were generated using NEB Next® Ultra™ DNA Library Prep Kit for Illumina (NEB, USA) following manufacturer’s recommendations and index codes were added. The library quality was assessed on the Qubit@ 2.0 Fluorometer (Thermo Scientific) and Agilent Bioanalyzer 2100 system. (vii) Sequencing data processing: PCR was performed by specific primers connecting with barcodes. The construction of the DNA libraries was used NEBNext® Ultra™ IIDNA Library Prep Kit (Cat No. E7645) and the processes of end repairing, adding A to tails, purification and Amplicon was sequenced on Illumina paired-end platform (Novaseq6000 platform PE250) to generate 250 bp paired-end raw reads, and then merged and pre-treated to obtain clean tags. The chimeric sequences in clean tags were detected and removed to obtain the effective tags which can be used for subsequent analysis.

### Statistical analyses

#### Performance and digestibility

The Statistical Analysis System (SAS Institute. 2010. Version 9.1.3) was used to conduct all statistical analyses. Means, standard deviations and pooled standard errors (SEM) were calculated for each variable. Data were tested for normality using univariate procedure of SAS. Data were analyzed with one-way ANOVA implemented in the GLM procedure of SAS, that included the effects of treatment and replicate (r = 1–16). Each replicate was considered as the experimental unit. As the mortality rate and number of dirty eggs were very low, these data were transformed by using a natural logarithm (ln) function [ln(y) = Log(y + 10)] to correct heterogeneity of variance and produce an approximately normally distributed data set. The log–log transformed data were analyzed with one-way ANOVA implemented in the proc GLM procedure of SAS. For the classification of the eggs upon size, the PROC FREQ procedure (SAS Institute, 2010, Version 9.1.3) was used to determine the frequency and percentage of individual egg category within treatment. The Chi-Square test was used to find out the significance effect of treatment on each class.

#### Beyond performance: Microbiome characterization

In order to analyze the species diversity within samples, all Effective Tags were clustered by Uparse software (Uparse v7.0.1001, http://drive5.com/uparse/) [27]. OTUs (Operational Taxonomic Units) were conducted at 97% and 95% sequence similarity by default. To confirm differences in the abundances of individual taxonomy between the two groups, Metastats software was utilized. LEfSe was used for the quantitative analysis of biomarkers within different groups. This method was designed to analyze data in which the number of species is much higher than the number of samples and to provide biological class explanations to establish statistical significance, biological consistency, and effect-size estimation of predicted biomarkers.

## Results

### Xylanase product

**Fig 1:**
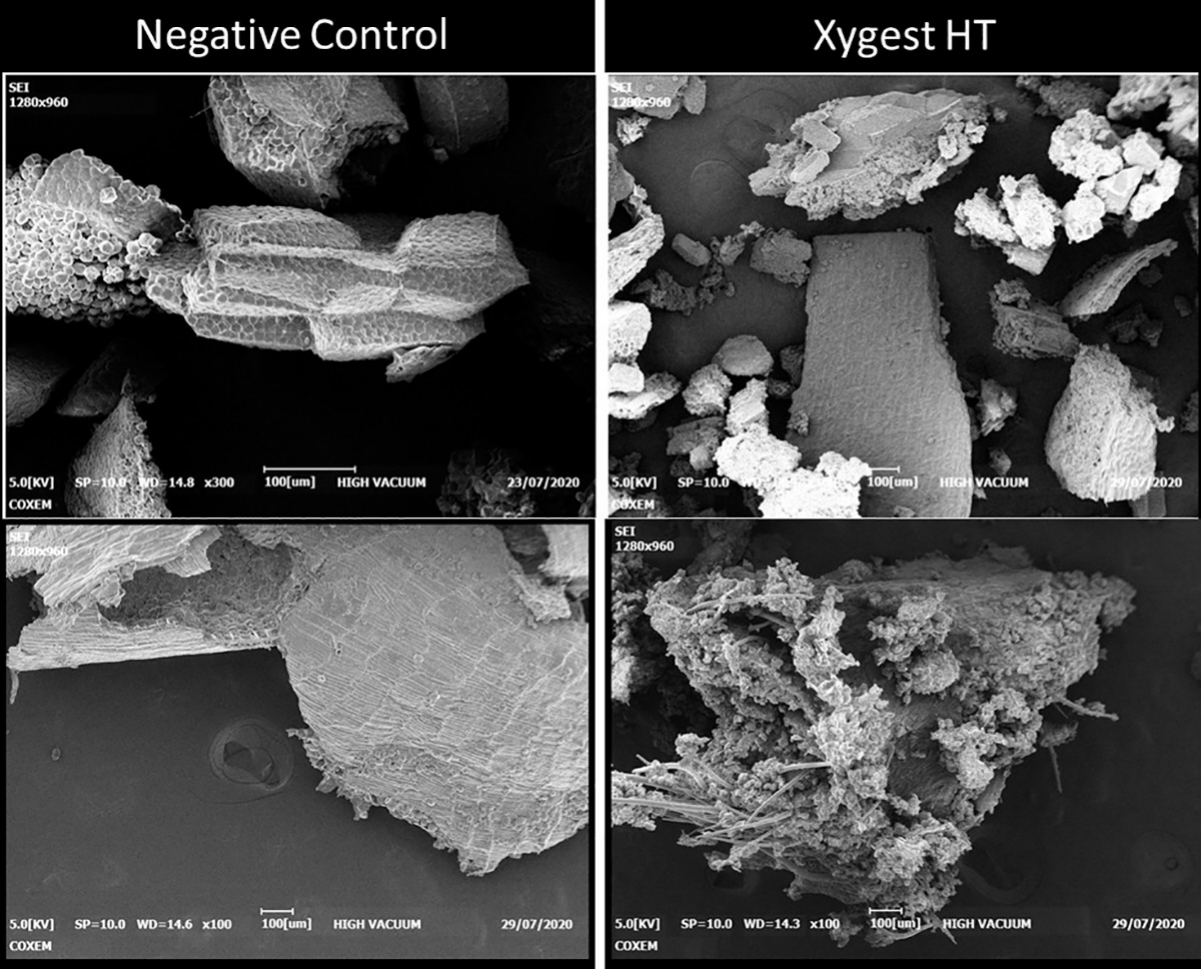
Scanning electronic visualisation of wheat from the negative control (no Xygest HT exposure) versus wheat exposed to Xygest HT.

A selection of the SEM captured photos is presented below (Fig 1). The SEM visualized micro structural differences between xylanase treated and untreated wheat particles. The particles in the untreated (negative control) wheat appeared to be densely compacted compared with loose structure in wheat exposed to the xylanase. The SEM showed also deep channels and vacuoles through the matrix of xylanase treated wheat, while the untreated wheat appeared with intact architecture (Fig 1).

### Performance

**Fig 2:**
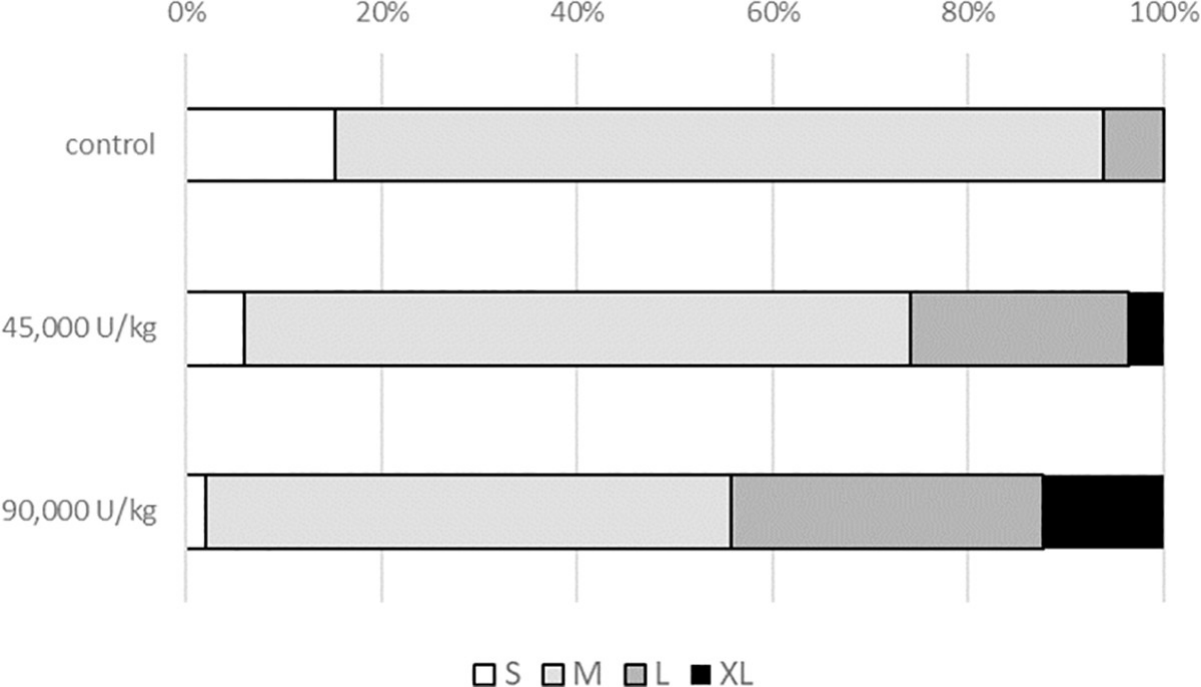
At week 12, eggs were commercially classified based on egg weights. All eggs laid in a 24-hour period were collected, weighed individually and classified commercially (S, M, L, XL), where S < 52.5 g; 52.5 g≤ M < 62.5 g: 62.5 g ≤ L< 72.5 g; XL > 72.5 g. The total number of eggs classified is 621.

There were no treatment effects on mortality. All performance data are presented in Tables 5–7. The lowest dosage, 45,000 U/kg, significantly increased average egg weight and improved feed efficiency compared to the control treatment (P<0.05). Egg quality parameters were significantly improved in the experiment. More of the eggs produced by 45,000 U/kg and 90,000 U/kg supplemented birds were in the L and XL classes compared to the control treatment (Fig 2). In addition, at 12 weeks of the study, the 45,000 and 90,000 U/kg treatments exhibited an increase in Haugh units and albumin heights (P<0.05). Furthermore, in the same period, the 90,000 U/kg group significantly changed yolk color compared to the control group (P<0.05).

**Table 5:** Performance characteristics of laying hens fed the control diets and diets containing xylanase at the different dosages for the whole trial period (84 days).

Treatment	Average egg weight (g)	Laying percentage (%)	Egg mass (g)	Feed intake (g/hen/day)	Feed efficiency (g feed/g egg)	Feed efficiency (kg feed / dozen eggs)
Control	55.58 c	88.24 b	49.05 c	109.92 a	2.26 a	1.50 a
45,000 U/kg	56.80 b	90.03 ab	51.14 b	108.43 ab	2.14 b	1.45 b
90,000 U/kg	58.00 a	90.70 a	52.61 a	106.51 bc	2.04 c	1.42 b
SEM	0.19	0.5	0.37	0.57	0.019	0.011

Values are means. Values within the same column that differ significantly (P<0.05) are indicated by different letters. SEM = pooled standard error of the mean.

**Table 6:** Egg quality in laying hens fed the control diets and diets containing xylanase.

	Treatment	Albumin Height (mm)	Haugh Unit	Yolk Color
Pre-experimental (14 days before start experiment)	Control	7.1	87.1	5.2
	45,000 U/kg	7.2	88.6	5.1
	90,000 U/kg	7.1	88.6	4.7
	SEM	0.15	0.84	0.22
Week 4	Control	6.9	84.8	4.7
	45,000 U/kg	7.2	85.2	4.5
	90,000 U/kg	7.4	85.6	4.7
	SEM	0.19	0.20	0.25
Week 8	Control	6.8 c	83.8	4.5
	45,000 U/kg	7.0 b	84.0	4.5
	90,000 U/kg	7.3 a	85.1	4.5
	SEM	0.14	0.91	0.24
Week 12	Control	6.9 c	83.1 c	5.2 b
	45,000 U/kg	7.3 b	85.7 b	5.6 ab
	90,000 U/kg	7.5 a	86.6 a	5.8 a
	SEM	0.15	0.92	0.17

Values are means at 4 weeks, 8 weeks and 12 weeks of the trial.

Values within the same column that differ significantly (P<0.05) are indicated by different letters. The total number of eggs analyzed is 144. SEM = pooled standard error of the mean.

**Table 7:** Apparent digestibility in laying hens fed the control diets and diets containing xylanase.

Treatment	Dry Matter Digestibility %	Organic Matter Digestibility %	Crude Protein Digestibility %	Nitrogen Retention g/kg	AME	AMEn	Gross Energy	Crude Fat Digestibility %	Crude Fibre Digestibility %
					kcal/kg	kcal/kg	Digestibility %		
Control	68.61 b	75.13 b	82.56 c	23.12 c	2,590.78 b	2,400.77 b	70.20 b	77.57 b	14.64 b
45,000 U/kg	70.41 ab	77.67 a	85.15 b	23.89 b	2,669.75 ab	2,473.34 a	72.29 ab	80.54 ab	16.97 ab
90,000 U/kg	71.33 a	78.45 a	87.94 a	24.64 a	2,684.93 a	2,482.39 a	72.71 a	81.64 a	23.24 a
SEM	0.56	0.41	0.64	0.18	21.28	20.12	0.58	0.99	2.48

Values are means. Values within the same column that differ significantly (P<0.05) are indicated by different letters. SEM = pooled standard error of the mean.

Crude Protein digestibility values have been corrected for uric acid nitrogen in the excreta.

Compared with the control, the apparent digestibility of OM and crude protein was drastically improved in the 45,000 U/kg treatment group (P<0.05). However, it is important to note that as uric acid levels were not measured in this study, improvements in N digestibility and AMEn are only apparent improvements. Furthermore, apparent GE and crude fat digestibility were also improved significantly in the 90,000 U/kg treatment. The AME values numerically increased by 3% in the 45,000 U/kg treatment and significantly increased by 4% in the 90,000 U/kg treatment, compared with the control.

### Beyond performance: Microbiome characterization

**Fig 3:**
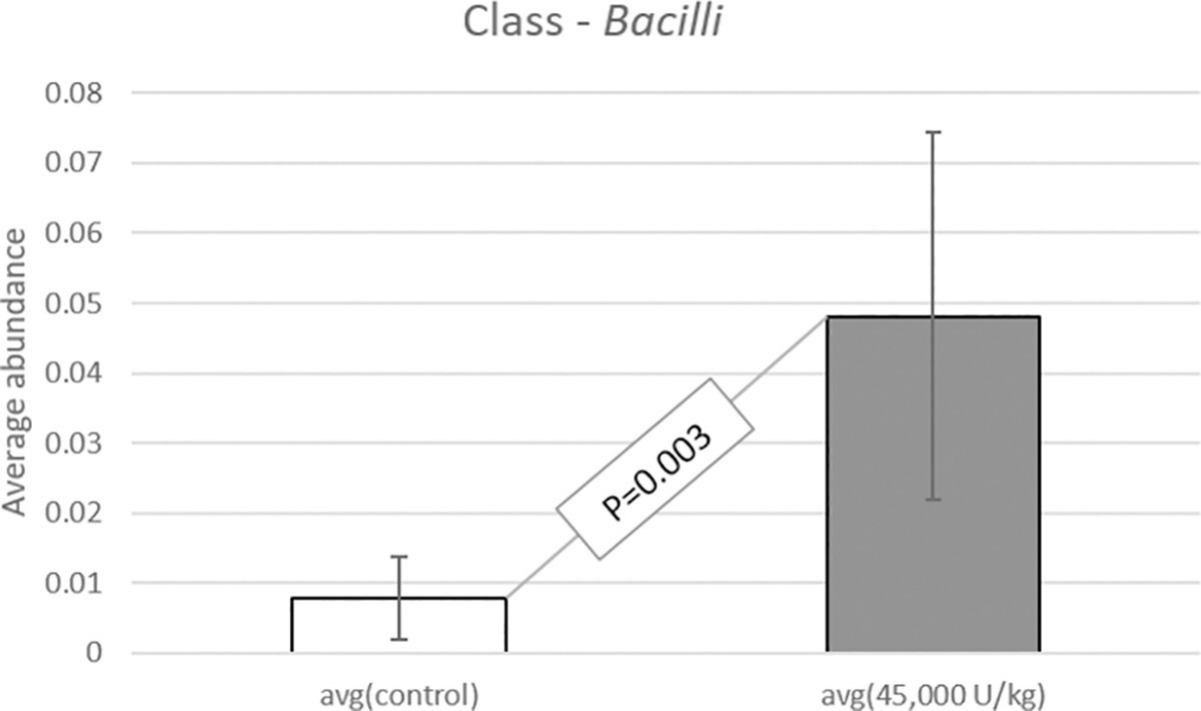
T-test comparison between groups based on “class”. Only the classes displaying a significantly different abundance between treatments are presented (P<0.05).

**Fig 4:**
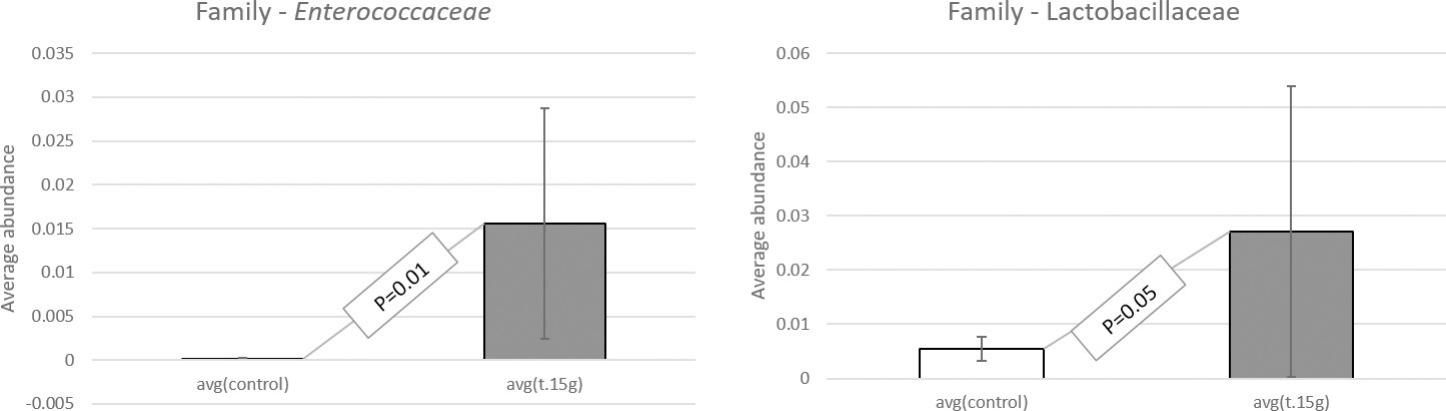
T-test comparison between groups based on “family”. Only the families displaying a significantly different abundance between treatments are presented (P<0.05).

**Fig 5:**
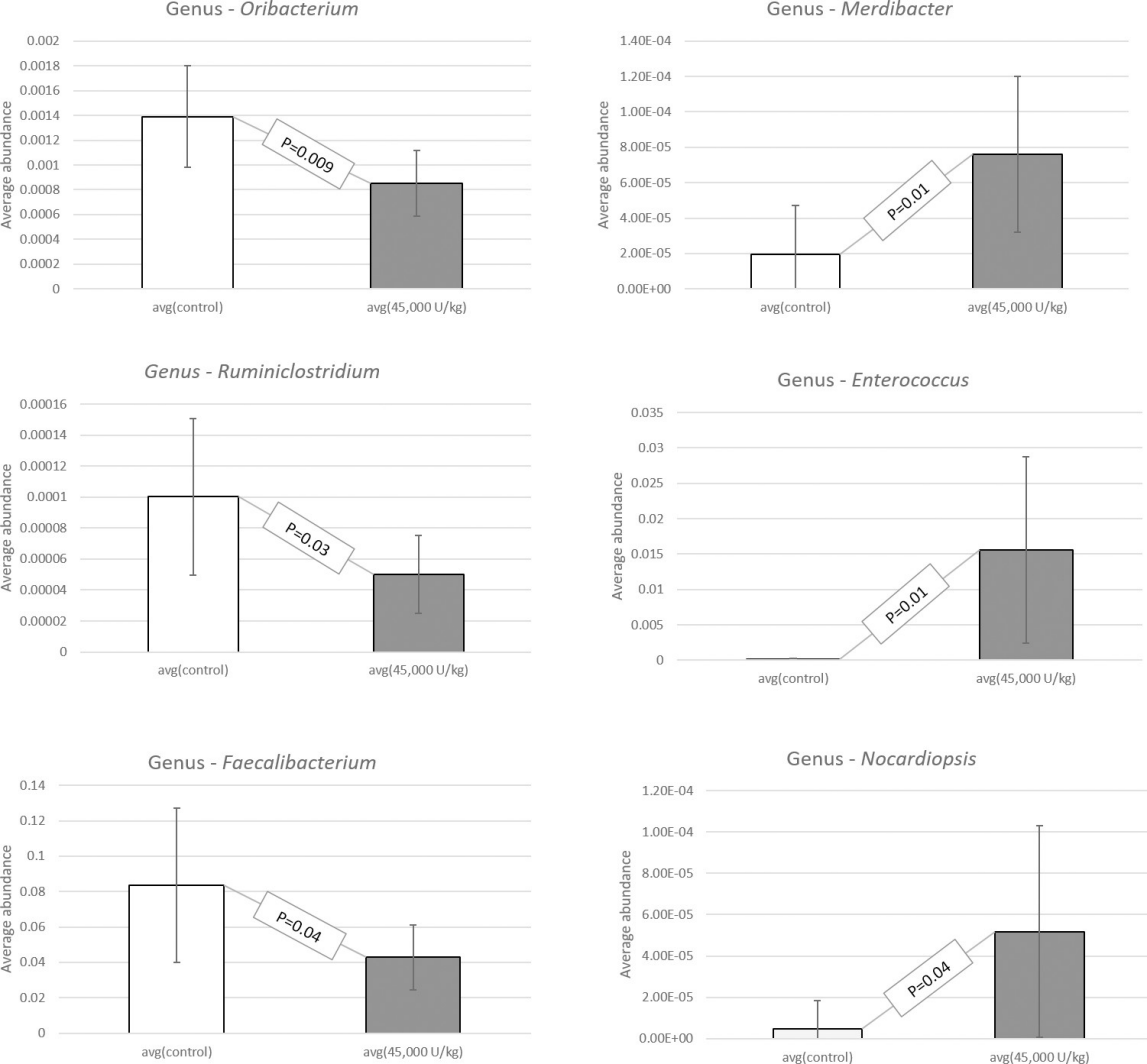
T-test comparison between groups based on “genus”. Only the genera displaying a significantly different abundance between treatments are presented (P<0.05).

**Fig 6:**
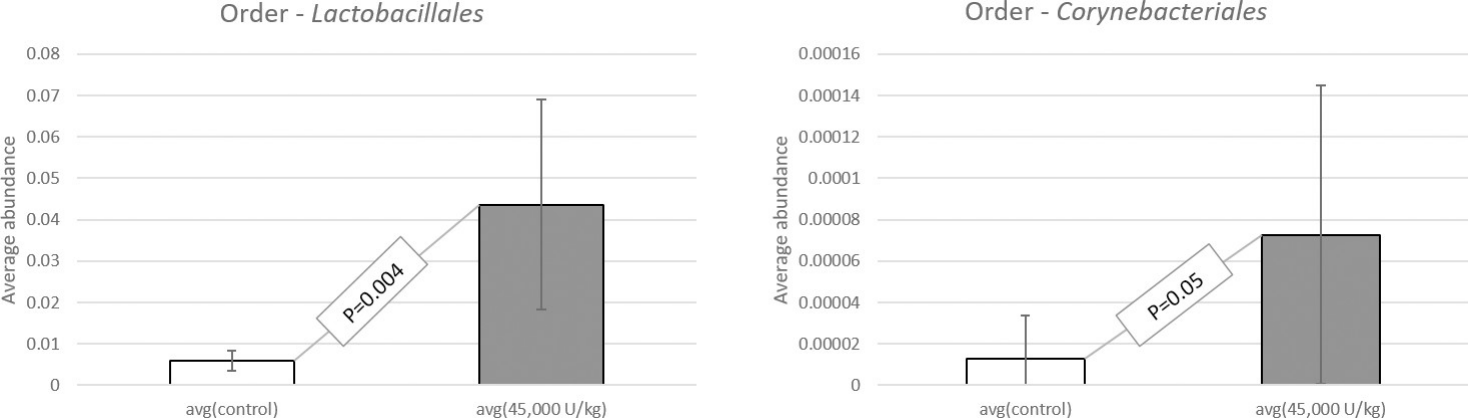
T-test comparison between groups based on “order”. Only the orders displaying a significantly different abundance between treatments are presented (P<0.05).

**Fig 7:**
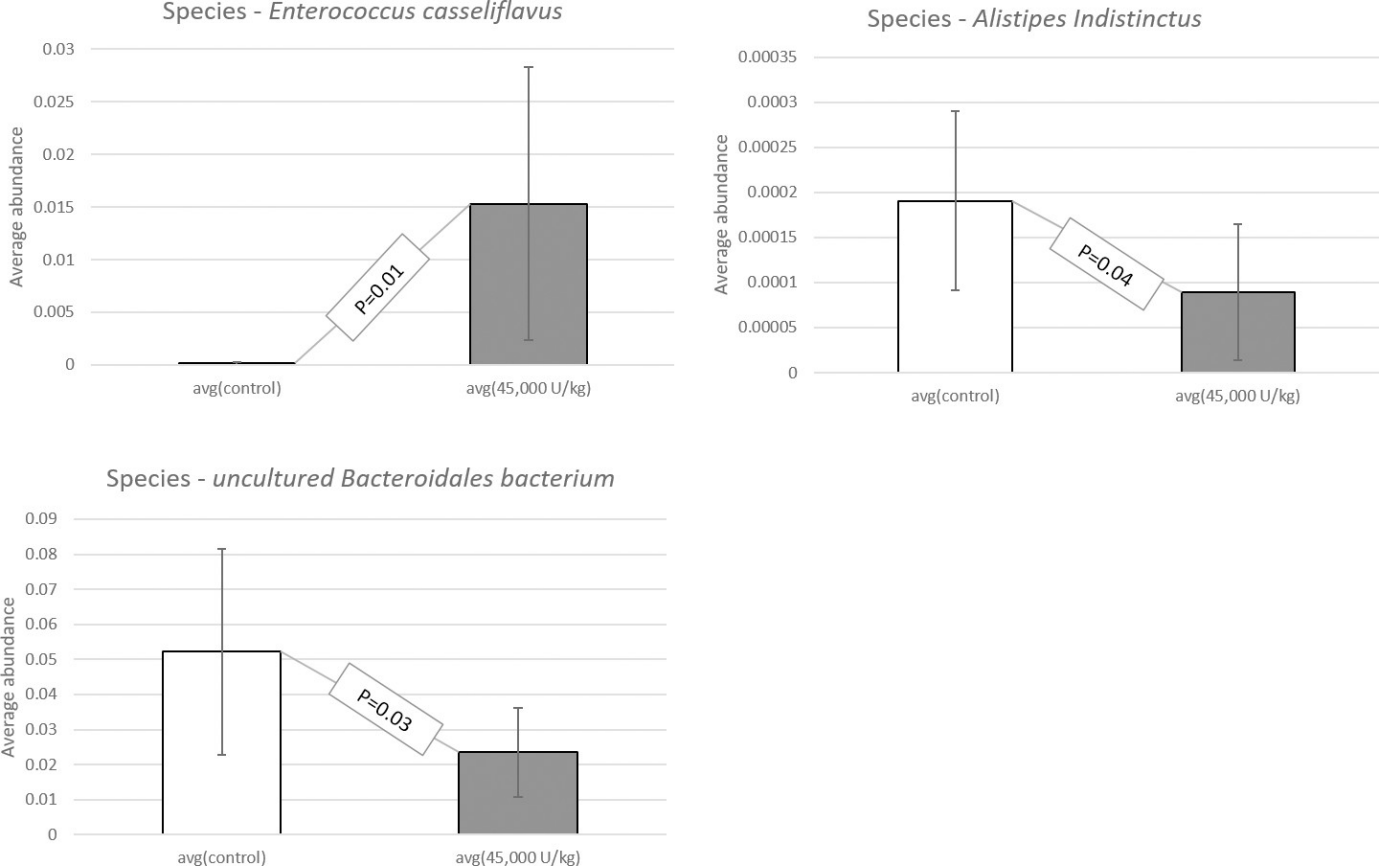
T-test comparison between groups based on “species”. Only the species displaying a significantly different abundance between treatments are presented (P<0.05).

When studying the sequence variation in the 16S rRNA gene in order to characterize diverse microbial communities of the cecal content, a significant increase in beneficial probiotic bacteria was observed when adding 45,000 U/kg xylanase to the diet of laying hens. All data have been deposited at the SRA database and are available under accession number: BioProject ID PRJNA725805. Data were screened for statistical differences between the two treatments and the following figures show the outcome of the t-test focusing on class (Fig 3), family (Fig 4), genus (Fig 5), order (Fig 6), and species (Fig 7). The bacterial population from the *Bacilli* class was 6 times more abundant in the 45,000 U/kg compared to the control group. For the family, t-test showed that *Enterococcaceae* (122 times) and *Lactobacillaceae* (5 times) were significantly more abundant in the xylanase-treated group compared to the controls. Three bacterial genera were more present in the xylanase-treated group compared to the control; *Merdibacter* (4 times increase), *Enterococcus* (122 times increase) and *Nocardiopsis* (11-fold increase). By contrast, three genera were decreased in the xylanase-treated group compared to the control; *Oribacterium* (2-fold decrease), *Ruminiclostridium* (2-fold decrease) and *Faecalibacterium* (2-fold decrease). When characterizing bacterial orders, there was a significant increase in *Lactobacillales* (7-fold) and the *Corynebacteriales* (6-fold) orders in the xylanase-treated group compared to the control. Finally, for the species, a higher abundance (122-fold increase) has been observed for the *Enterococcus casseliflavius* in the xylanase-treated group compared to the control, whereas a 2 times decreased abundance has been observed for the *uncultured Bacteroidales* species and for *Alistipes indistinctus*.

**Fig 8:**
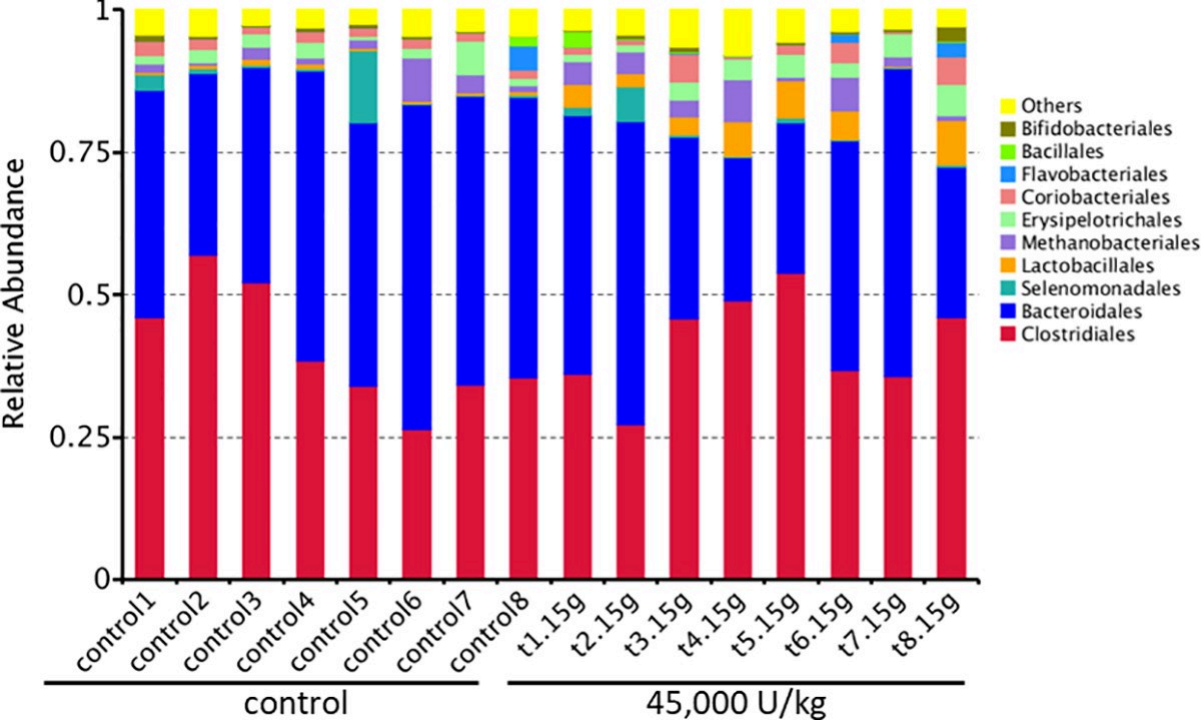
Top 10 genera abundance across the different samples.

When documenting the relative abundance of the top 10 genera in Fig 8, the image depicts that the *Lactobacillus* population is enriched in the layers fed with the 45,000 U/kg dosage compared to the control animals.

**Fig 9:**
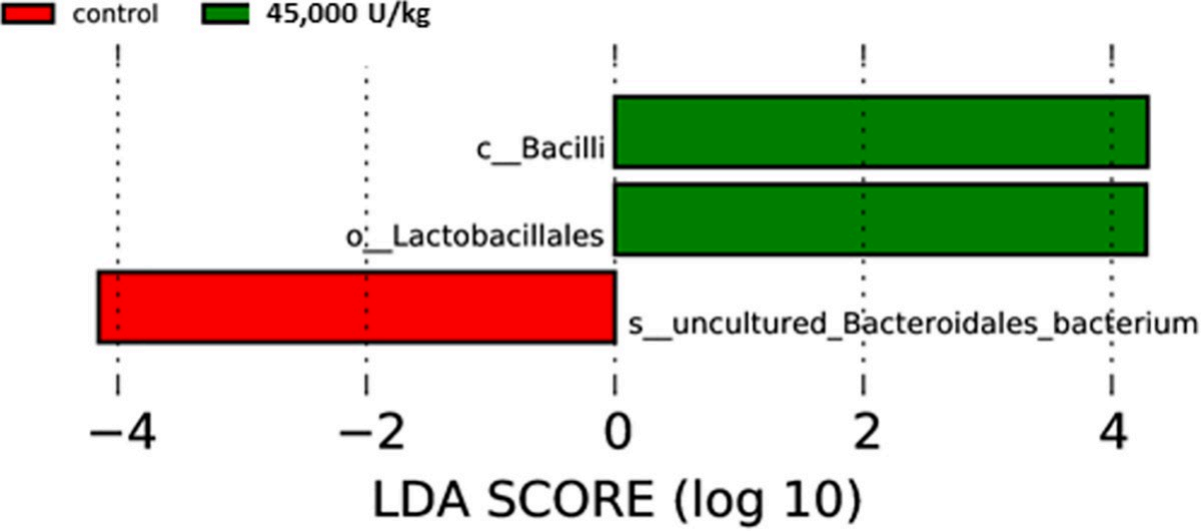
LDA analysis bar chart comparing the two treatment groups. The latter chart highlights potential biomarkers. In this chart, c_ indicates “class”, o_ indicates “order”, s_ indicates “species”. The LDA scores are shown as the results of LEfSe analysis for evaluating of biomarkers with statistically difference among groups. The histogram of the LDA scores represents species (biomarker) whose abundance shows significant differences among groups. The selecting criteria is that LDA scores are larger than the set threshold (4 set by default). The length of each bin, namely the LDA score, represents the effect size (the extent to which a biomarker can explain the differentiating phenotypes among groups).

Linear discriminant analysis (LDA) (Fig 9) reveals information on potential biomarkers. LDA is closely related to analysis of variance (ANOVA) and regression analysis, which presents a dependent variable as a linear combination of other features or measurements [28].

**Fig 10:**
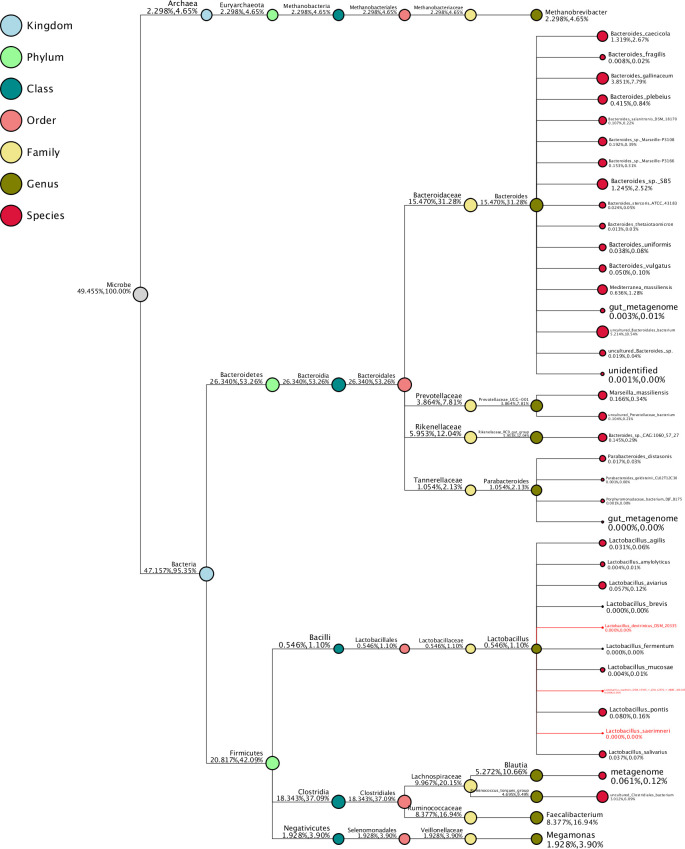
Taxonomy tree of the control group. Top 10 genera in high relative abundance by default were selected to make the taxonomy tree. The species name in red refers to the lower abundance, close to zero. The size of circles represents the relative abundance of species. The first number below the taxonomic name represents the percentage in the whole taxon, while the second number represents the percentage in the selected taxon.

**Fig 11:**
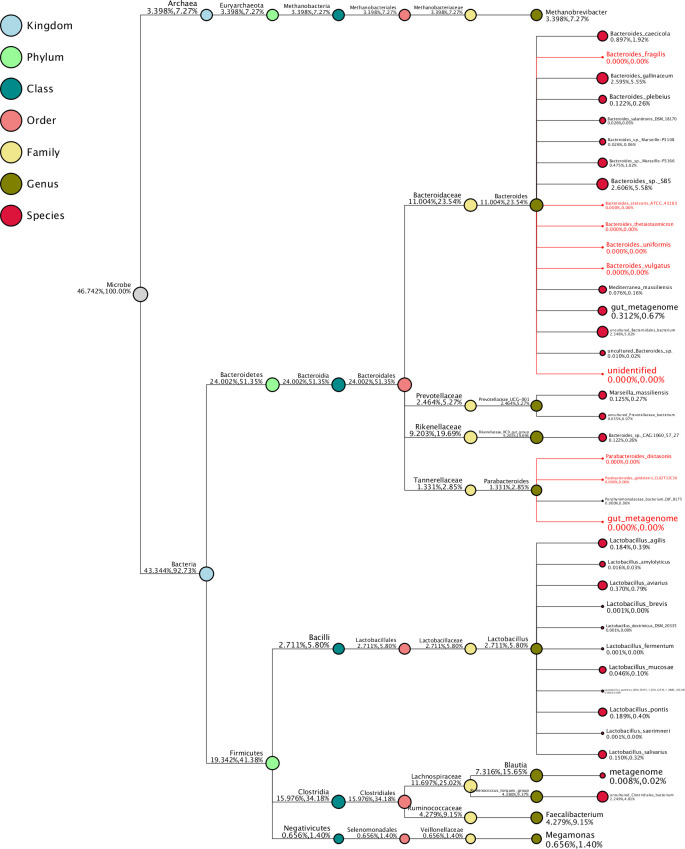
Taxonomy tree of the 45,000 U/kg group. Top 10 genera in high relative abundance by default were selected to make the taxonomy tree. The species name in red refers to the lower abundance, close to zero. The size of circles represents the relative abundance of species. The first number below the taxonomic name represents the percentage in the whole taxon, while the second number represents the percentage in the selected taxon.

In Figs 10 and 11, a taxonomy tree is shown for the control group and the 45,000 U/kg group, respectively. The size of the red circles represents the relative abundance of the species.

## Discussion

Exogenous enzymes can hydrolyze NSP, which affects the digestibility and utilization of nutrients [9]. In this study, we proposed that the xylanase is also able to modulate the laying hen microbiome beneficially, and thus can exert a prebiotic effect. Kogut et al. [15] documented on how the gastrointestinal tract is rich in microbial biodiversity, playing home to ≥500 phylotypes or ∼1 million bacterial genes, which equates to 40–50 times the size in the chicken genome. Manipulating the microbiota using the xylanase enzyme could be a promising approach to improve poultry gut health.

Overall, for each of the zootechnical parameters measured, hens fed the control diet performed worse than those fed diets supplemented with the xylanase. The significant increase in all performance parameters of xylanase supplemented birds supports the use of this xylanase for feed efficiency in layer diets high in NSPs. The positive impacts of the xylanase on laying performance appeared in the late phase and prove the efficiency of the xylanase to maintain a normal egg laying rate or even a higher performance compared to the average standards reported by the laying hen strain used in the present study [29]. These results corroborate the findings of Mathlouthi *et al*. [30] who found that the enzyme can improve egg production, egg mass and feed conversion in layers fed wheat-barley-based diets. Mirzae *et al*. [31] also reported increased egg production, egg mass, and feed efficiency, and decreased digesta viscosity due to xylanase supplementation in diets containing wheat. The improvement in laying performance is likely driven by the significant improvements on energy utilization and nutrient–including protein—availability in response to xylanase addition, as reflected in the digestibility data.

Interestingly, the change in yolk color was slight but significant. Several researchers have previously reported the absence of xylanase effects on yolk color [32–34]. The improvement in yolk color obtained in the last experimental phase can be explained by the increase in apparent crude fat digestibility in these treatments. Carotenoids are usually lipophilic substances, and thus they are stored well in fats [35]. Birds consuming the 90,000 U/kg treatment were able to utilize 5.0% more digestible fat than those fed the control diet. Considering that fat is subsequently responsible for transporting pigmentation to the egg yolk, these results imply more fat-soluble pigments were present in the yolk of 90,000 U/kg supplemented hens.

With regards to the microbiome characterisation, the observed stimulation of the *Lactobacillales* order is clearly beneficial. The immunomodulatory activities of *Lactobacillus* probiotic bacteria have been well documented in mammals [36]. It has been shown that *Lactobacillus* culture beneficially impacts immune response and cytokines in chickens [37–39] and, moreover, can limit *Salmonella* colonization [40]. The species *Enterococcus casseliflavus*, being a probiotic [41], was also enriched in the 45,000 U/kg group compared to the controls. Based on the LDA analysis, the *Bacillus* class and the *Lactobacillales* order being enriched seems to be a marker for the xylanase-treated animals. The latter again supports the hypothesis that xylanase addition beneficially impacts on the bacterial population in the ceca of the laying hens. In the taxonomic tree (Fig 11), one can see that the *Bacteroides* and *Parabacteroides* genera have a very low relative abundance in the 45,000 U/kg group, which might be indicative for good gut health [42].

The prebiotic effect of xylanase achieved in this study is proposed to be caused by several mechanisms. First, this prebiotic effect could be due to XOS emerged through cleaving of xylan backbone [9]. The XOS work as nutrient substrate for beneficial bacteria and have been reported as promising prebiotics [43]. There are several reports documenting on how XOS can improve gut health in poultry [44, 45]. Although, in the present study, the change in flow of individual fiber fractions at ileal and total tract levels was not measured, further research on this topic would clearly elucidate this mode of action.

Another possible mechanism might be that the prebiotic effect is achieved via the release of cell wall-entrapped oligosaccharides. Some nutrients such as starch, oligosaccharides and protein are trapped within the insoluble fibrous cell wall where poultry are unable to access these trapped nutrients [46]. The SEM images (Fig 1) demonstrate that the addition of the xylanase used in this study can cause the enzymatic hydrolysis of the nutrients. Fig 1 revealed a dense skeleton structure in the negative control while wheat particles appeared to be fragmented in the xylanase exposed wheat. This complex skeleton could hold the releasing of entrapped nutrients and thus reduce digestibility of control treatment. In contrast, the fragmented architecture in the xylanase exposed wheat may be involved in the positive effects of nutrients release.

A third mechanism could be that xylanase exerts a stimbiotic effect. The term stimbiotic has been introduced recently and is defined as non-digestible but fermentable additives that stimulate fiber fermentability but at a dose that is too low that the stimbiotic itself could contribute in a meaningful manner to volatile fatty acid [47]. The stimbiotic effects include the speeding up development of a fiber-fermenting microbiome that gives a kickstart to the microbiome to ferment the fiber [48]. The prebiotic and stimbiotic mechanisms of xylanases have been recently described in pigs [49].

In conclusion, the xylanase assessed in this study not only positively impacts performance but might also exert a prebiotic effect by stimulating the growth of several beneficial bacteria, while, by contrast, reducing the abundance of pathogens. This microbiome flexibility in response to xylanase addition could be an attractive target for intervention.
